# Supplementary material for: MIXTURE of human expertise and deep learning—developing an explainable model for predicting pathological diagnosis and survival in patients with interstitial lung disease
Source: Mod Pathol. 2022 Feb 23;35(8):1083–91. doi: 10.1038/s41379-022-01025-7 (PMC9314248; doi:10.1038/s41379-022-01025-7)
Supplement: Supplementary file 1 — Supplemental data [file 41379_2022_1025_MOESM1_ESM.pdf]

Supplemental data

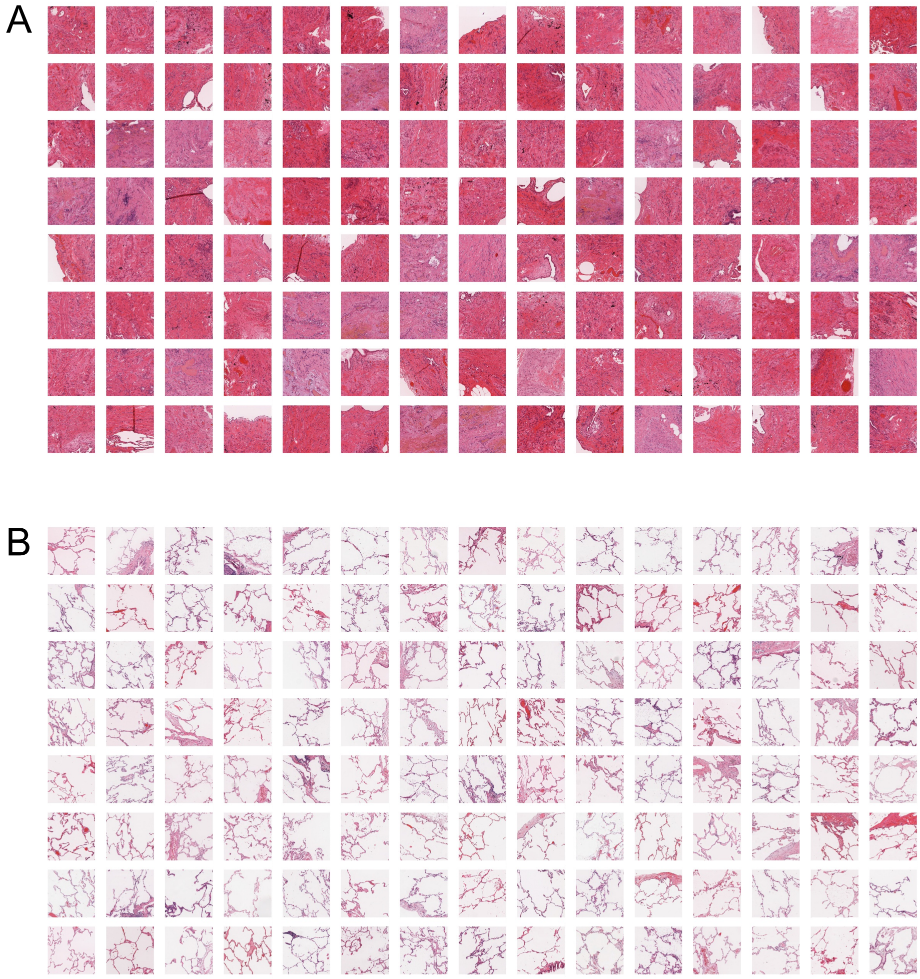

Figure. S 1: **Example of montage.** The tiles cut from each magnification were converted into feature vectors by ElEx and clustered based on the similarity. In order to demonstrate the overview of each cluster, 120 tiles were randomly selected from each cluster and a montage was created. These examples are all tiles extracted at 5x magnification from different cases; A is classified as acellular fibrosis and B as complete normal.

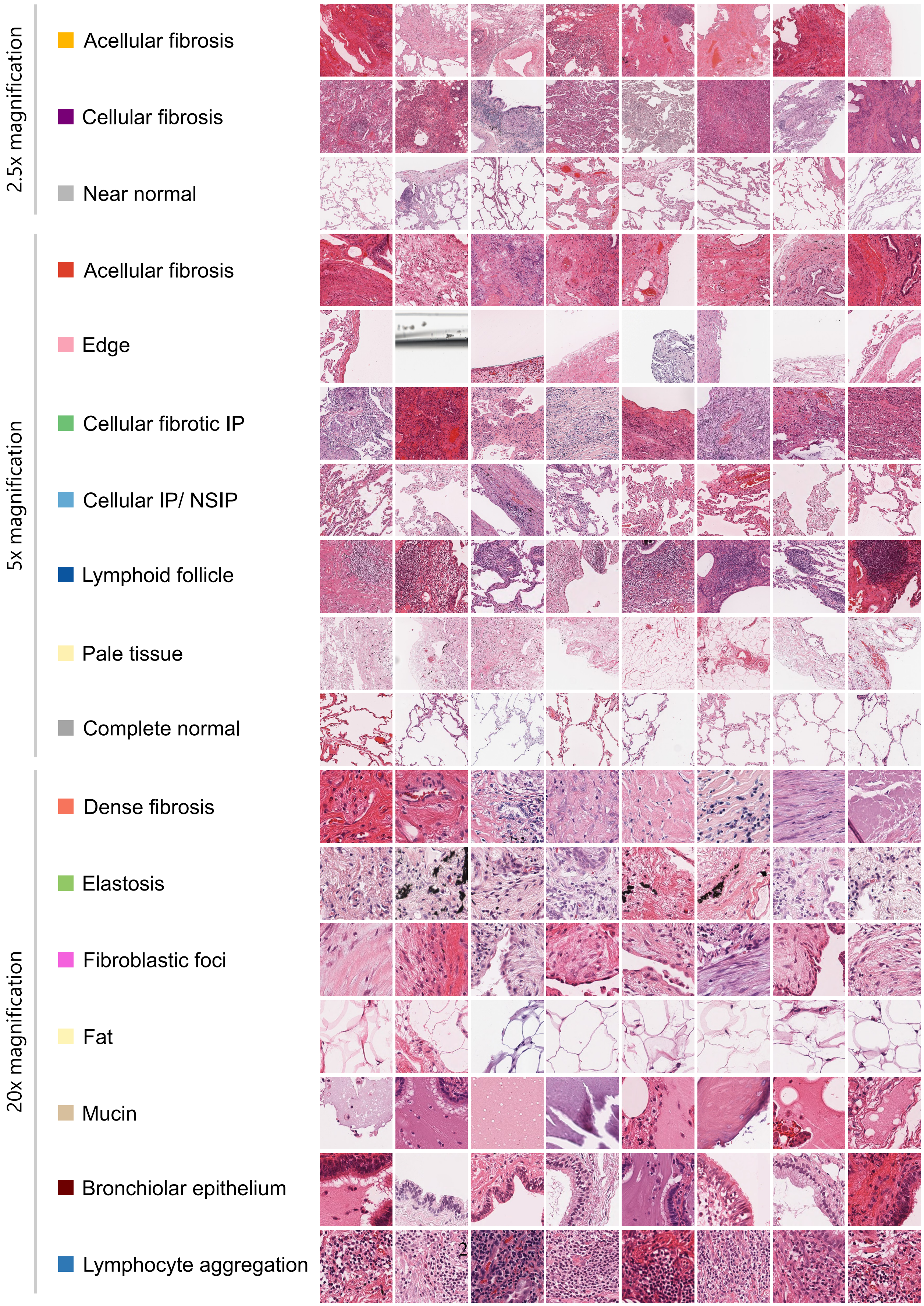

Figure. S 2: **Example of tiles for each class.**

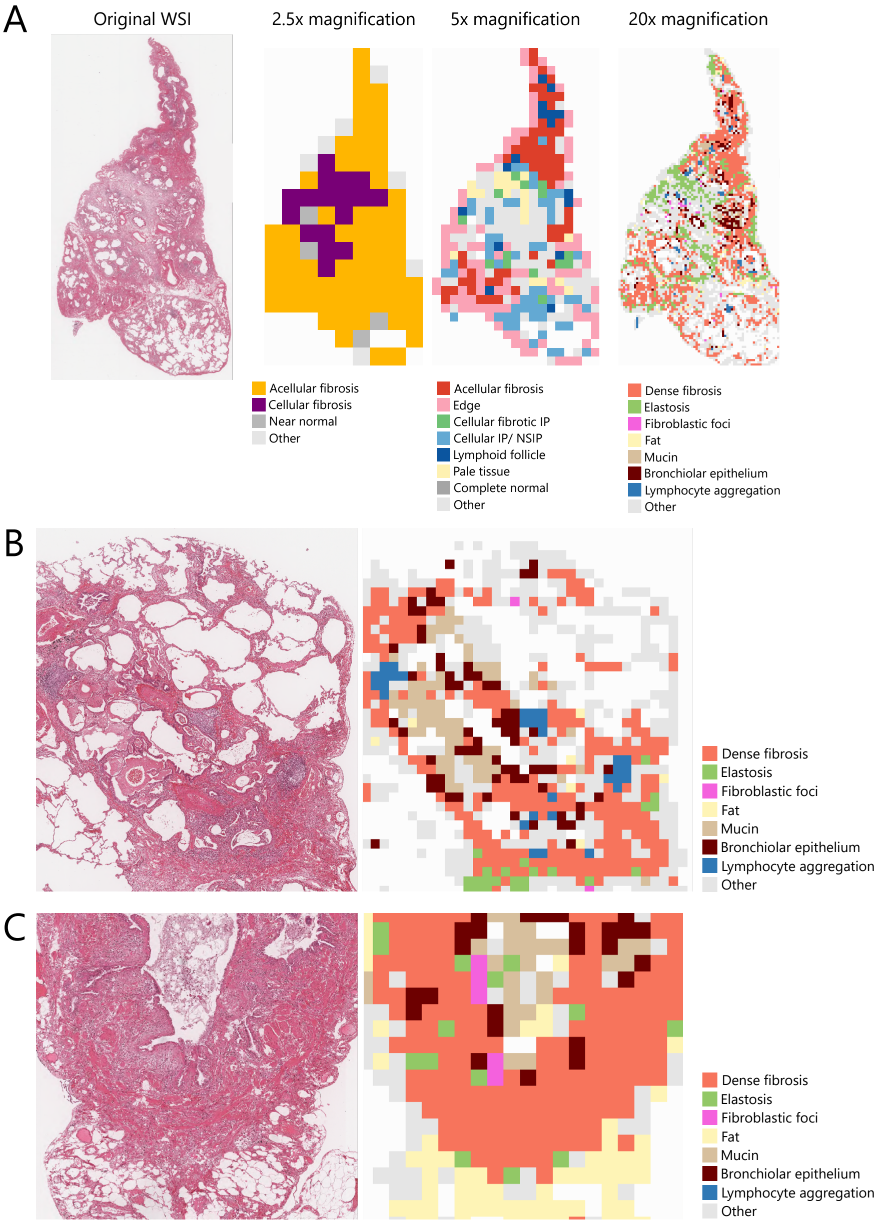

Figure. S 3: **Additional examples of feature extraction.** A: A case of interstitial pneumonia related to systemic sclerosis with a histological diagnosis of ”probable UIP”. B, C: Preview of 20x feature extractor with high magnification

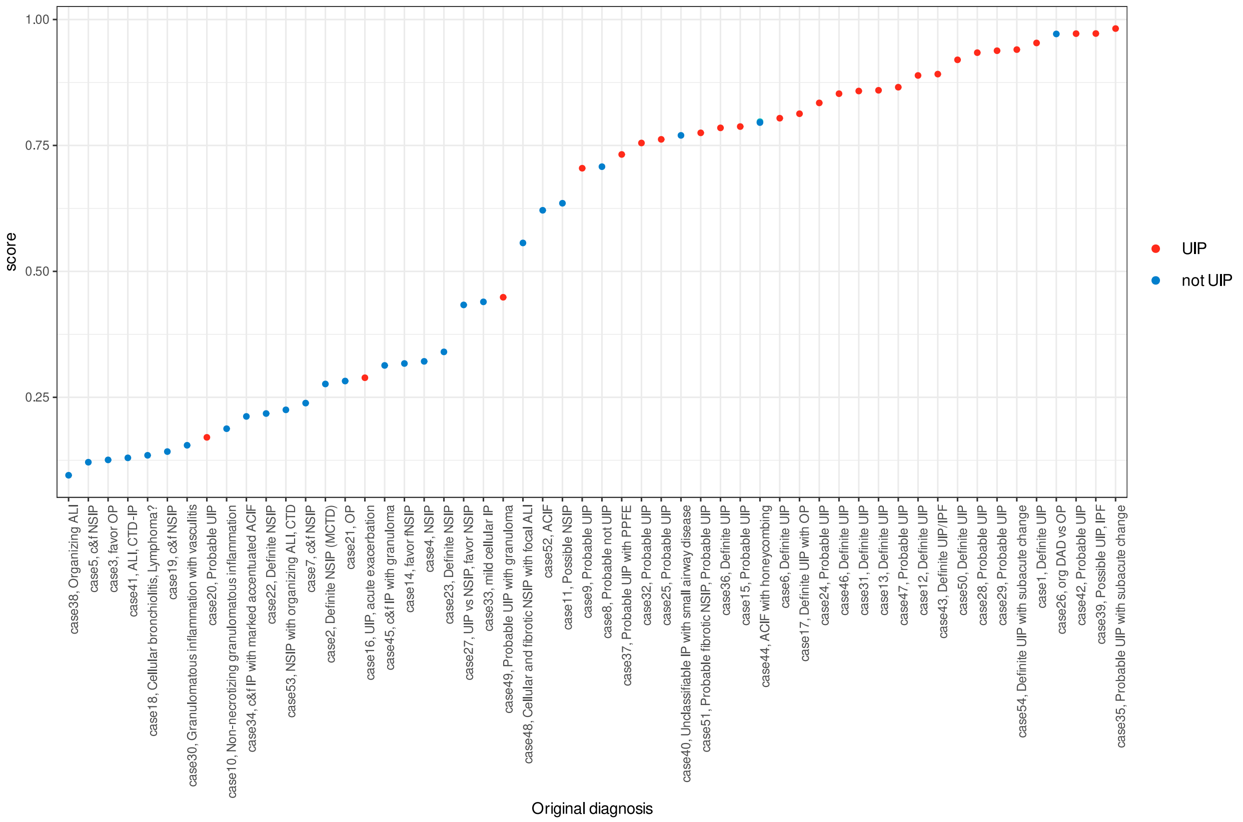

Figure. S 4: **Score of random forest regressor and the actual histopathological diagnosis.** The x-axis shows the actual pathological diagnosis for each case, and the y-axis shows the predicted UIP score by the random forest 5x model. Cases regarded as UIP from its pathology report are shown in orange, and not UIPs are shown in blue.

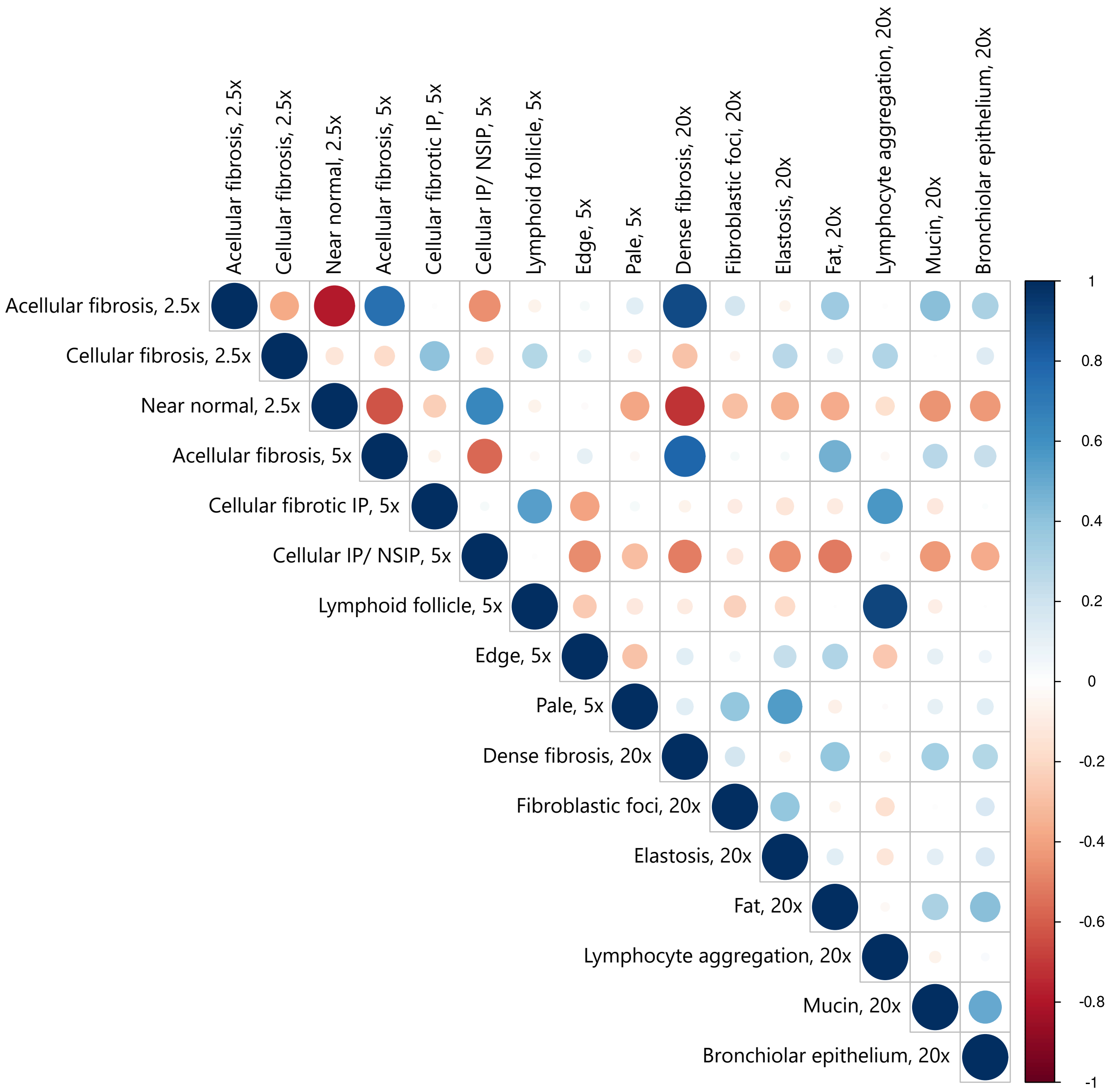

Figure. S 5: **Quantitative correlation of extracted findings.** Because similar findings were extracted for each magnification, pairs of findings with correlated occurrences were found in the same case. There is a positive correlation between acellular fibrosis extracted at 2.5x and acellular fibrosis extracted at 5x. Similarly, there is a very strong positive correlation between lymphatic follicles extracted at 5x and lymphocytes extracted at 20x.

Table. S 1: **Feature importance of 20x model**

Findings	Importance
Dense fibrosis, 20x	3.540
Fibroblastic foci, 20x	2.875
Elastosis, 20x	3.685
Fat, 20x	6.099
Lymphocyte aggregation, 20x	2.610
Mucin, 20x	2.765
Bronchiolar epithelium, 20x	5.218

Table. S 2: **Feature importance of 2.5x+5x model**

Findings	Importance
Acellular fibrosis, 2.5x	1.802
Cellular fibrosis, 2.5x	2.112
Near Normal, 2.5x	1.902
Acellular fibrosis, 5x	3.473
Cellular and fibrotic IP, 5x	2.724
Cellular IP/ cNSIP, 5x	7.102
Lymphoid follicle, 5x	2.234
Edge, 5x	3.598
Pale, 5x	2.335

IP: interstitial pneumonia, cNSIP: cellular non-specific interstitial pneumonia

Table. S 3: **Feature importance of 5x+20x model**

Findings	Importance
Acellular fibrosis, 5x	2.223
Cellular fibrotic IP, 5x	2.183
Cellular IP/ NSIP, 5x	5.175
Lymphoid follicle, 5x	1.336
Edge, 5x	2.124
Pale, 5x	1.644
Dense fibrosis, 20x	1.492
Fibroblastic foci, 20x	1.200
Elastosis, 20x	1.326
Fat, 20x	3.357
Lymphocyte aggregation, 20x	1.003
Mucin, 20x	1.518
Bronchiolar epithelium, 20x	3.067

IP: interstitial pneumonia, cNSIP: cellular non-specific interstitial pneumonia

Table. S 4: **Feature importance of 2.5x+5x+20x model.**

Findings	Importance
Acellular fibrosis, 2.5x	0.751
Cellular fibrosis, 2.5x	1.412
Near normal, 2.5x	0.767
Acellular fibrosis, 5x	2.030
Cellular fibrotic IP, 5x	1.850
Cellular IP/ NSIP, 5x	4.690
Lymphoid follicle, 5x	0.994
Edge, 5x	1.834
Pale, 5x	1.362
Dense fibrosis, 20x	1.356
Fibroblastic foci, 20x	0.959
Elastosis, 20x	1.282
Fat, 20x	3.415
Lymphocyte aggregation, 20x	0.899
Mucous, 20x	1.247
Bronchiolar epithelium, 20x	2.929

IP: interstitial pneumonia, NSIP: non-specific interstitial pneumonia

Table. S 5: **UIP prediction by support vector machine algorithm**

	AUC	95% CI
Proposed model		
2.5x	0.87	0.77 - 0.97
5x	0.89	0.79 - 0.97
20x	0.9	0.81 - 0.98
2.5x + 5x	0.86	0.76 - 0.97
5x + 20x	0.88	0.79 - 0.97
2.5x + 20x	0.89	0.80 - 0.98
2.5x + 5x + 20x	0.86	0.77 - 0.96
Non-integrated model		
k=4	0.47	0.31 - 0.63
k=8	0.55	0.39 - 0.71
k=10	0.56	0.40 - 0.72
k=20	0.51	0.35 - 0.67
k=30	0.47	0.31 - 0.63
k=50	0.61	0.46 - 0.77
k=80	0.61	0.46 - 0.76

AUC: area under the receiver operator characteristic curve, CI: confidence interval

Table. S 6: **Subgroup analysis consisting of cases diagnosed as UIP by pathologists. In the Cox proportional hazards model, the amount of fibroblastic foci was confirmed to be a risk factor.**

	HR	95% CI	p value
Cellular fibrosis	1.13	0.69 – 1.84	0.6339
Cellular IP/ NSIP	0.81	0.33 – 1.97	0.6402
Edge	1.22	0.76 – 1.98	0.4134
Dense fibrosis	1.30	0.80 – 2.12	0.2932
Fibroblastic focus	1.67	1.13 – 2.46	0.0010
Elastosis	1.09	0.65 – 1.81	0.7528
Fat	1.06	0.75 – 1.50	0.7427
Lymphocyte aggregation	1.27	0.60 – 2.71	0.5302
Mucin	1.19	0.80 – 1.76	0.3907
Bronchiolar epithelium	0.59	0.37 – 0.94	0.0268

IP: interstitial pneumonia, NSIP: non-specific interstitial pneumonia, HR: hazard ratio, CI: confidence interval

Table. S 7: **Subgroup analysis consisting of cases diagnosed as not UIP by pathologists. In the Cox proportional hazards model, the aggregated lymphocytes were identified as a risk factor.**

	HR	95% CI	p value
Cellular fibrosis	0.19	0.028 – 1.26	0.0855
Cellular IP/ NSIP	0.83	0.25 – 2.76	0.7602
Edge	1.52	0.68 – 3.97	0.3958
Dense fibrosis	1.59	0.44 – 5.67	0.4789
Fibroblastic focus	1.87	0.69 – 5.06	0.2182
Elastosis	2.16	0.57 – 8.17	0.2579
Fat	0.56	0.29 – 10.77	0.7013
Lymphocyte aggregation	2.87	1.03 – 7.97	0.0426
Mucin	1.24	2.30 – 5.17	0.7639
Bronchiolar epithelium	1.02	0.32 – 3.25	0.9779

IP: interstitial pneumonia, NSIP: non-specific interstitial pneumonia, HR: hazard ratio, CI: confidence interval
